# Targeting intracellular pathways in idiopathic inflammatory myopathies: A narrative review

**DOI:** 10.3389/fmed.2023.1158768

**Published:** 2023-03-13

**Authors:** Gaetano La Rocca, Francesco Ferro, Chiara Baldini, Alessandro Libra, Domenico Sambataro, Michele Colaci, Lorenzo Malatino, Stefano Palmucci, Carlo Vancheri, Gianluca Sambataro

**Affiliations:** ^1^Rheumatology Unit, Department of Clinical and Experimental Medicine, University of Pisa, Pisa, Italy; ^2^Regional Referral Centre for Rare Lung Disease, Azienda Ospedaliero Universitaria Policlinico “G. Rodolico-San Marco”, University of Catania, Catania, Italy; ^3^Artroreuma S.R.L., Rheumatology Outpatient Clinic, Catania, Italy; ^4^Internal Medicine Unit, Rheumatology Clinic, Azienda Ospedaliera per l’Emergenza Cannizzaro, University of Catania, Catania, Italy; ^5^Department of Medical Surgical Sciences and Advanced Technologies “GF Ingrassia”, University Hospital Policlinico “G. Rodolico-San Marco”, Catania, Italy

**Keywords:** JAK/STAT 1, 2, 3, dermatomyositis, antisynthetase syndrome, Baricitinib, Tofacitinib

## Abstract

In recent decades, several pieces of evidence have drawn greater attention to the topic of innate immunity, in particular, interferon (IFN) and Interleukin 6 in the pathogenesis of idiopathic inflammatory myopathies (IIM). Both of these molecules transduce their signal through a receptor coupled with Janus kinases (JAK)/signal transducer and activator of transcription proteins (STAT). In this review, we discuss the role of the JAK/STAT pathway in IIM, evaluate a possible therapeutic role for JAK inhibitors in this group of diseases, focusing on those with the strongest IFN signature (dermatomyositis and antisynthetase syndrome).

## Introduction

Idiopathic inflammatory myopathies (IIM) represent a group of systemic autoimmune disorders sharing striated muscles as their preferred target, but potentially involving various organ systems including the skin, lungs, joints and gastrointestinal tract (1). Specifically, interstitial lung disease (ILD) is a common manifestation, representing one of the main causes of mortality (2).

In recent decades it has become progressively more evident that IIM cannot be bundled into a single entity. In fact, IIM patients exhibit heterogeneous clinical phenotypes, histologic findings and peripheral autoantibody repertoires, pointing to the existence of different underlying pathogenetic pathways (3).

In 1975, Bohan and Peter distinguished between polymyositis and dermatomyositis relying on the presence or absence of a typical skin rash, while the most recent 2017 EULAR/ACR classification criteria allow for the stratification of IIM patients into 6 major subgroups based on clinical and pathological features: polymyositis (PM), inclusion body myositis (IBM), amyopathic dermatomyositis (ADM), dermatomyositis (DM), Juvenile dermatomyositis (JDM) and Juvenile myositis other than JDM (4–6).

Moreover, the discovery of new myositis specific antibodies (MSA), strongly associated with the clinical features of IIM patients (7), will probably lead to new classification criteria discriminating distinct entities such as antisynthetase syndrome (ASS) and immune-mediated necrotizing myopathies (IMNM), in the spirit of a modern phenotype-based approach. Indeed, various authors reported an overdiagnosis of PM according to the classical Bohan and Peter criteria, and a recent cluster analysis on a large French cohort showed that PM patients were more correctly classified in other subgroups, mainly IMNM and ASS (8, 9). Accordingly, nowadays the existence of a pure PM entity is debated.

Therapeutic options for IIM patients are limited, and mortality rates are still especially high for some IIM subsets, such as anti-MDA5+ IIM (10). Steroids continue to be a therapeutic cornerstone, with concerns about their chronic use causing potential side effects, especially considering that steroid sparing drugs are currently less efficacious compared with other connective tissue diseases (11).

In view of this, in this era of precision medicine, dissecting the exact pathogenetic mechanisms underlying IIM is a fundamental pre-requisite in order to forge a new path toward effective, patient- and phenotype-tailored target therapies.

Given recent evidence from the successful employment of Janus kinase inhibitors (JAKi) in the treatment of diverse interferon (IFN)-mediated autoinflammatory processes, the present review will discuss the involvement of Janus kinases (JAK) mediated pathways in IIM and their potential as a therapeutic target.

We specifically focus on IIM subsets with a strong IFN signature, namely DM, ASS and anti-MDA5+ ADM.

### DM, ASS, and ADM

Dermatomyositis and ASS are distinguished clinically from PM mainly by the association of myositis with a typical inflammatory skin involvement. DM patients usually present serum autoantibodies such as anti-Mi2, anti-TIF1gamma, anti-NXP2, and anti-SAE which are correlated with specific clinical features, risk for associated cancer and prognosis (12). In contrast, the hallmark of ASS is the association of serum anti-aminoacyl tRNA synthetase autoantibodies with the triad of myositis, arthritis and ILD (13). Indeed, at disease onset, a significant proportion of ASS patients show only one item of the classic triad: the risk of developing other signs seems to be associated with the presence of a specific antibody (14). Finally, ADM is defined by the presence of typical vasculitic skin lesions, with little (hypomyopathic DM) or no clinical or histologic muscle involvement. Anti-MDA5 autoantibodies are detected in at least 50% of patients in ADM cohorts, a prevalence which is probably influenced by the laboratory techniques employed, and are strongly associated with a high risk of rapidly progressing ILD (RP-ILD) (15). A clusterisation of ADM patients into 3 main phenotypic groups with different RP-ILD risk and prognosis has been proposed, with some differences between European and Asiatic cohorts (10, 16).

### The JAK/STAT pathway

The term JAK/signal transducer and activator of transcription proteins (STAT) refers to molecules mediating a widespread intracellular signaling pathway, physiologically involved in haematopoiesis, adipogenesis, apoptosis and immune response, influencing both innate and adaptive immunity (17). However, the aberrant activation of this pathway is associated with the development of autoimmune diseases and carcinogenesis (18). The discovery of this pathway has led to great advances in knowledge of the pathogenesis of several diseases, and more importantly, has highlighted a promising therapeutic target.

Janus kinases-coupled cytokine receptors undergo dimerisation after binding by their extracellular ligands. Dimerisation induces the phosphorylation of JAKs, which in turn phosphorylate STAT. This latter activated form of STAT translocates into the nucleus regulating gene transcription. This represents the canonical process of JAK/STAT activation. The pathway can also be activated in a non-canonical way, by oxidative stress-induced tyrosine kinases, 7-elix membrane receptors or cellular hypertonicity (19).

There are currently 4 known forms of JAK: JAK1, JAK2, JAK3, and TYK2. JAK1, JAK2, and TYK2 are ubiquitous. JAK1 phosphorylation is induced by both IFN-I (mainly α/β), IFN-II (γ) and cytokines belonging to the interleukin (IL) 2, IL6 and IL10 families. JAK2 is activated by similar ligands, but also by hormones, while TYK2 mainly mediates the signaling of IFNs, IL6 and IL10. Finally, JAK3 is mostly involved in the negative selection and production of lymphocytes, and is therefore only present in bone marrow and lymphoid tissue and is activated by cytokines belonging to the IL2 family (20).

The STAT family includes 7 proteins: STAT1, STAT2, STAT3, STAT4, STAT5a, STAT5b, and STAT6. STAT1 is activated by all of the IFN, IL2, and IL6 families, tumour necrosis factor (TNF) and other chemokines. Its role is to favor apoptosis, inhibit cell growth, and regulate cell differentiation. STAT1 also plays a role in the regulation of the immune system, transducing the signal of major histocompatibility complexes (MHC) after antigen presentation and allowing the development of B cells. STAT2, STAT3, and STAT4 are activated by IFN-I, exerting different actions.

Signal transducer and activator of transcription proteins (STAT2) regulates the immune response of macrophages and T cells, with an antiviral effect. STAT3 also mediates IL6 and IL10 signaling, resulting in activation of the Th17 response and inhibition of apoptosis. STAT4 is also phosphorylated by receptors recognizing IL12 family cytokines, favoring a Th1 response. STAT5a and STAT5b are activated by IL2 family cytokines and prolactin, with a role in apoptosis, lactation and production of immune cells. Finally, STAT6 is mainly activated by IL4 and IL13. It is crucial for Th2 differentiation, and the proliferation and maturation of B cells, as well as for the expression of MHC II and IgE (20).

Numerous cytokines and hormones transduce signals through the JAK-STAT pathway, but with different strengths, and with the ability to activate in multiple ways. For example, IFN-I is classically associated with a strong activation of STAT1 and a weaker activation of STAT3 and 4. Other cytokines with opposite functions are able to act in the same way (for example IL6 and IL10 on STAT3). The final effect of an immune stimulus depends on the composition and relative quantities of the cytokine milieu released, the duration and intensity of JAK/STAT signaling, the types of STAT proteins coupled with JAKs and the cell types involved in the process (21).

The idea of interfering with JAK/STAT mediated immune processes led to the synthesis of small molecules acting as JAKi, initially approved for the treatment of rheumatoid arthritis (RA). First generation JAKi are non-selective. The first two molecules approved were Tofacitinib, a pan-inhibitor of JAKs, with strong activity against JAK1 and JAK3, and minor activity against JAK2 and TYK2, and Baricitinib, exerting a significant inhibitory action on JAK1 and JAK2, moderate activity against TYK2 and minimal activity against JAK3 (22). New generation JAKis display greater selectivity for JAK1, thus potentially limiting the hematological side effects related to interference with JAK3 (23). Up to the present moment, the only JAK1 inhibitors approved for RA treatment, Upadacitinib and Filgotinib, have shown great efficacy coupled with an acceptable safety profile (24, 25).

In light of the ability of JAKis to inhibit production of both Th1 and Th2 cytokines, particularly IL6, thus also impairing the polarization of Th17 lymphocytes, they are currently being tested for the treatment of a broad range of inflammatory disorders (26).

### Current pathogenetic models in DM, ADM, and ASS

#### JAK-STAT pathway in IIM

The JAK1-STAT1/STAT3 axis is pivotal in the physiology of skeletal muscles.

It promotes myogenesis by exerting a potent antidifferentiation action on myoblasts’ premature differentiation, and premature formation of myotubes (27). In contrast, activation of the JAK2-STAT2/STAT3 axis leads to the opposite effects.

Therefore, STAT3 is located downstream on a physiological axis potentially able to cause both muscle growth and wasting. IL6 plays a pivotal role in both pathways, as its receptor is associated with the two axes (28). IFN-γ can interact with JAK1 and JAK2, but is also able to activate STAT3 inducing muscle wasting *via* NFκb, independently of IL6 (29). This data highlights the “double-edged sword” role of STAT3 in skeletal muscle physiology.

Signal transducer and activator of transcription proteins (STAT3) is also crucial in skin homeostasis. It is phosphorylated by JAK1, JAK2, and TYK2, regulating the migration of keratinocytes (30). Physiologically, STAT3 is necessary in wound healing and protection from ultraviolet rays, but if constantly activated is associated with the development of cutaneous rashes in lupus erythematosus, psoriasis, and atopic dermatitis, as well as with carcinogenesis (30, 31).

Finally, in normal lung tissue, STAT1, STAT5a, and STAT5b promote inflammation, while STAT2 and STAT6, respectively, have a pro and an anti-apoptotic effect. STAT4 is involved in the response to IFN-γ for the regulation of immune response, and STAT3 in the transduction of the IL6 pathway, favoring cell proliferation (32).

The principal role in ILD patients seems to be played by the JAK2/STAT3 axis. STAT 3 is overexpressed in lung macrophages, endothelial cells, myofibroblasts and neutrophils in idiopathic pulmonary fibrosis (IPF) and systemic sclerosis patients, allowing the deposition of extracellular matrix leading to fibrosis (33). Finally, transforming growth factor β (TGFβ) is recognized by JAK2-coupled receptors that are highly expressed in fibroblasts, hyperplastic alveolar epithelial type II cells, and in the small pulmonary arteries of IPF patients (34).

The JAK2/STAT3 pathway therefore mediates TGFβ and IL6 induced pro-fibrotic and pro-inflammatory effects leading to ILD in connective tissue diseases (33). In a recent *in vitro* assay, Baricitinib was able to inhibit JAK2/STAT3 and therefore prevent IL6 induced epithelial-mesenchymal transition (35). Moreover, Ruxolitinib (another JAK1/JAK2 inhibitor) was successfully employed in the treatment of ILD secondary to a STAT3 gain of function mutation (36).

#### The prominent role of IFNs and IL-6

On a pathologic level, DM is characterized by mononuclear cells infiltrates with predominant T CD4+ lymphocytes and prevalent perivascular distribution, leading to perifascicular myofiber atrophy in muscle samples and vasculitic changes in skin biopsies. This key contribution of adaptive immunity to the pathogenesis of the disease is further supported by known genetic associations with human leukocyte antigen (HLA) alleles, the presence of specific serum autoantibodies, and the proven efficacy of immunosuppressive strategies targeting cellular (calcineurin inhibitors) and humoral (anti-CD20 monoclonal antibodies) autoimmunity (37, 38).

However, the main focus of translational research has progressively shifted toward the role of innate immunity players in initiating the autoimmune process (39).

Perimysial atrophic fibers in DM biopsies typically show upregulation of MHC-I molecules, whose expression is known to be induced by IFN-I (40). Actually, IFN-α/β induced genes were found to be highly expressed in muscle biopsies of DM patients compared to controls, PM, and IBM, together with the presence of MxA, an IFN-I induced protein (41). The IFN-I signature was also found in peripheral blood and skin biopsies, longitudinally correlating with disease activity in DM and ADM patients. Since then, the presence of an IFN-I signature was also identified in peripheral blood and skin samples from DM and ADM patients (42, 43).

In contrast to the predominant type 1 IFN axis activation observed in DM, gene expression analysis of ASS muscle biopsies identified a type 2 IFN signature (44, 45). This is in line with the expression of IFN-II induced MHC-II molecules on the membranes of necrotic perifascicular myofibers in ASS, a rare occurrence in DM muscle samples (46).

Both IFN-I and IFN-II are widely produced by innate immunity phagocytes: mainly plasmacytoid dendritic cells (pDC) for IFN-I, monocytes-macrophages and NK lymphocytes for IFN-II. IFN production is stimulated by the non-specific binding of molecules shared between phylogenetically correlated pathogens to pattern recognition receptors (PRRs) expressed by innate immunity cells. However, activation of PRRs, including membrane, cytoplasmic and endosomal toll-like receptors (TLRs) can also be triggered by endogenous DNA or RNA particles released by apoptotic cells (or neutrophils undergoing NETosis) either alone or complexed with autoantibodies (47). In this regard, it is notable that most autoantigens targeted by DM associated autoantibodies are DNA- or RNA-complexed proteins (48).

Immature muscle precursors are an alternative source of IFN-I. They also have an enhanced expression of autoantigens, possibly providing a further local stimulus for the production of IFN through PRRs (49, 50). Finally, the presence of pDC was reported in DM and JDM patients’ muscles and may be related to *in loco* production of IFN-I in muscle tissue (51).

The role of IFN-I in IIM was confirmed in the trial involving Sifalimumab, an anti-IFN-α antibody. Treated patients showed suppression of IFN signature associated with improved strength, however, a subsequent trial emphasized excessive side effects (NCT00979654) (52).

Along with IFNs, recent studies have highlighted the involvement of IL-6 in the physiopathology of IIM related organ damage. IL-6 is a cytokine with pleiotropic effects, physiologically involved in immune response and the hematopoietic, endocrine and nervous systems (53). IL-6 knockout mice were less prone to develop muscular inflammation in a murine model of myositis (54). More importantly, *in vivo* studies demonstrated that adult and JDM patients exhibit higher IL-6 levels compared to healthy controls, and IL-6 levels correlate with disease activity and an IFN-I signature (55–57).

Interestingly, DM, ASS, and anti-MDA5+ DM patients with ILD display higher IL-6 serum levels than patients without ILD (56, 58).

Indeed, IL-6 has been shown to exert pro-inflammatory and pro-fibrotic effects, by stimulating fibroblasts, both in preclinical and *in vivo* studies of IPF and non-IPF ILD patients (59).

Various case reports have highlighted the efficacy of Tocilizumab (an anti-IL6R) in the treatment of refractory DM, ASS and anti-MDA5+ ADM, both with chronic fibrosing- and RP-ILD (60–63). However, a recent phase IIb trial comparing Tocilizumab to a placebo in the treatment of DM and PM patients failed to meet its primary endpoint (64).

**FIGURE 1:**
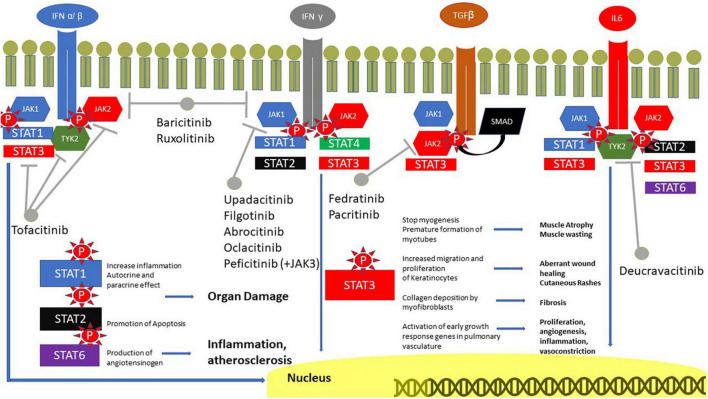
Role of JAK in IIM. IFN receptors are coupled with JAK1 and 2, IFN α/β and IL6 also with TYK2. TGFβ is coupled with SMAD, however, it can also phosphorylate JAK and JAK2. The signal is transmitted to the nucleus through STAT, with a great role of STAT3. STAT1 is involved in inflammation, STAT2 in the promotion of apoptosis and STAT3 in the production of angiotensinogen. JAK, Janus kinases; IFN, interferon; IL, interleukin; SMAD, suppressor of mothers against decapentaplegic; STAT, signal transducer and activator of transcription proteins; TGF, transforming growth factor.

In light of the aforementioned evidence pointing to the relevance of IFNs and IL-6 in the pathogenetic mechanisms underlying skin, muscle and lung damage in IIM, blocking their signaling with JAKi represents a promising therapeutic strategy that is currently being explored intensively (Figure 1).

### Targeting intracellular pathways in IIM with JAKi: Where are we now?

In the last few years, an impressive number of reports have highlighted Tofacitinib efficacy in diverse domains of DM patients with heterogeneous phenotypes (65).

In a small case series, 3 patients with refractory cutaneous DM were treated with Tofacitinib (either 5 mg bid or 10 mg bid, in monotherapy or combined with Hydroxychloroquine), improving their lesions according to the cutaneous dermatomyositis disease area and severity index (CDASI) activity score (66). In a subsequent open-label trial (STIR), 10 patients with mainly cutaneous refractory DM were treated with Tofacitinib. The primary endpoint of the international myositis assessment and clinical studies group (IMACS) definition of improvement was met for all patients and the CDASI showed a mean decrease of 66% from baseline (67). Last year, the results of the long-term extension of this study were published: notably 7/10 patients retained Tofacitinib therapy for a mean 1.2 years, demonstrating a sustained response without the reintroduction of steroids (68).

Regarding the muscular domain, according to a systematic review published last year, 15/16 (93.8%) DM patients with muscular involvement treated with JAKi experienced clinical and/or imaging improvement (69).

Moreover, numerous studies have reported on the beneficial effects of Tofacitinib in the management of anti-MDA5+ ADM related ILD. Particularly, 18 anti-MDA5+ patients with early stage ILD were treated with combined steroids and Tofacitinib in an open label trial, showing higher 6-month survival rates compared to historical controls treated conventionally (70, 71). Another retrospective study compared in a small cohort of anti-MDA5+ ADM the efficacy of Tofacitinib and Tacrolimus, proving a better outcome in patients exposed to JAKi (72). Survival rate was low in both groups, notably, however, a great proportion of the enrolled patients were classifiable as RP-ILD, a condition known to portend a very poor prognosis, especially in anti-MDA5+ ADM patients (73).

Baricitinib also showed rapid beneficial effects in several case series including refractory adult and JDM (74–76). In 2022, two open label trials met their primary endpoint consisting of a clinically significant improvement of the CDASI activity score in DM patients treated with Baricitinib (77, 78).

Tofacitinib and Baricitinib also seem to be effective in calcinosis, as described in numerous real-life experiences (76, 79, 80).

Additional trials are ongoing, with the aim of investigating the efficacy of Baricitinib in both adult and JDM patients (NCT04208464, NCT05524311, NCT04972760, NCT05361109).

Conversely, only two case reports are available on the use of JAKis in ASS: Tofacitinib improved two ASS patients with an RP-ILD resembling ADM, though positive for anti-Jo1 and anti-EJ, respectively (81, 82).

Table 1 summarizes the current experiences and available evidence on JAKi in IIM.

**TABLE 1:** Published clinical trials on the use of JAK inhibitors in idiopathic inflammatory myopathies.

Molecule	Mechanism of action	Type of study	Number of patients	Results
Tofacitinib (66)	Pan-inhibitor	Open label trial	10 DM	Improvement of skin involvement according to IMACS and CDASI
Tofacitinib (70)	Pan-inhibitor	Open label clinical study	18 anti-MDA5 ADM	Improved survival in ILD
Tofacitinib (72)	Pan-inhibitor	Retrospective studies	26 anti-MDA5 ADM	Improved survival in ILD compared to tacrolimus
Baricitinib (77)	JAK1–2 inhibitor	Open label pilot study	12 DM	Improvement of skin involvement according to CDASI
Ruxolitinib (77)	JAK1–2 inhibitor	Open label pilot study	4 DM	Improvement of skin involvement according to CDASI
Baricitinib (78)	JAK1–2 inhibitor	Prospective, open label study	12 DM	Improvement of skin involvement according to CDASI

ADM, amyopathic dermatomyositis; CDASI, cutaneous dermatomyositis disease area and severity index; DM, dermatomyositis.

Besides the aforementioned JAKi, alternative approaches to target intracellular pathways in IIM are being explored. Brepocitinib, a dual TYK2 and JAK1 inhibitor, showed efficacy in preliminary studies and is currently being investigated in a phase III RCT on DM patients (NCT05437263). Similarly, TLRs are located upstream in the cascade leading to IFN hyperproduction and 2 different anti-TLR agents will be the object of phase II RCTs (NCT05650567) (83). Table 2 resumes recent and ongoing RCT investigating new promising molecules in IIM patients.

**TABLE 2:** Recent and ongoing trials investigating new molecules interfering with intracellular pathways in IIM.

Molecule	Mechanism of action	Type of trial	Patients	Primary outcome measure	Results
Brepocitinib (NCT05437263)	anti-JAK1/TYK2	Phase III, randomized, double-blind, placebo-controlled	DM	Total improvement score (TIS) at week 52	Ongoing
Enpatoran (NCT05650567)	TLR-7/8 antagonist	Phase IIa, randomized, parallel, double-blind, placebo controlled	DM, PM, ASS	- TIS at week 24; - Adverse events; - Clinically significant changes in laboratory, vital signs and ECG	Ongoing
IMO-8400 (83)	TLR7/8/9 antagonist	Phase II, randomized, double-blind, placebo-controlled	DM	Modified CDASIv2 activity score	Did not meet primary endpoint
Apremilast (84)	PDE4 inhibitor	Phase IIa, open-label, non-randomized controlled	DM	Overall response rate based on a 4 point decrease in CDASI at 3 months	Met primary endpoint
KZR-616 (NCT04033926)	Immunoproteasome inhibitor	Phase II, randomized, double-blind, placebo-controlled, crossover	DM, PM	Mean change in TIS	Ongoing
GLPG3667 (NCT05695950)	TYK2 inhibitor	Phase II, randomized, double-blind, placebo controlled	DM	Efficacy, safety, tolerability, pharmacokinetics	Not yet recruiting

### RP-ILD in IIM: Lesson learned from COVID-19 pandemic and future perspectives

An increasing amount of evidence suggests that RP-ILD in ADM patients is probably driven by IFN-γ axis upregulation, in line with the presence in their serum of biohumoural markers typically reflecting macrophagic hyperactivation, namely hyperferritinemia, high C Reactive Protein (CRP), peripheral cytopenia (especially lymphopenia), and high Lactic Dehydrogenase (LDH) and IL1 levels. Moreover, higher levels of these biomarkers have been associated with adverse lung and global outcomes in ADM subjects (85–88).

In view of this, important clues regarding the pathophysiology of ADM related RP-ILD come from experience acquired in the management of SARS-CoV-2 related severe interstitial pneumonia. Indeed, the severe hyperinflammatory phenotype of COVID-19 pneumonia shares striking biohumoural, clinical and histopathologic similarities with ADM related RP-ILD.

Wang et al. (89) also demonstrated that the presence of serum anti-MDA5 autoantibodies in COVID-19 patients represents a marker of severe disease and anti-MDA5 titres in severe COVID-19 patients correlate with mortality.

Furthermore, MDA5 is physiologically involved in the IFN-mediated response to viral infections and anti-MDA5 autoantibodies are thought to be directly pathogenic in ADM (90). Accordingly, plasma exchange and intravenous immunoglobulins showed efficacy in the treatment of both COVID-19 and anti-MDA5 ILD (91–93).

Of note, IFN-γ axis seems to be the main driver of inflammation and fibrosis also in SARS-CoV-2 pneumonia (94, 95).

As Baricitinib is the most effective available JAKi acting on JAK2 (31), it has been widely employed in clinical trials and real-life experience, proving to be efficacious in reducing the need for supplemental oxygen, as well as reducing mortality and the duration of hospitalization in intensive care units (96–101).

Based on these physiopathological considerations, we believe that in the near future, treatment with Baricitinib of both DM-related fibrosing ILD and ADM related RP-ILD may provide important advantages compared with Tofacitinib. Moreover, anti-MDA5+ RP-ILD patients still present dramatically high mortality rates due to progression of respiratory failure, despite exposure to aggressive combined conventional immunosuppressants. Therefore, current evidences probably justify early administration of JAKi as a first line treatment in these subset of patients, possibly combined with therapies targeting the humoral immune response (namely IVIg, PEX, and Rituximab) (102).

Serum biomarkers reflecting the hyperactivation of IFN-γ may not only serve as prognostic factors but also represent important clues when choosing the most appropriate JAKi agent.

Similarly, despite lack of studies exploring the potentialities of Upadacitinib and Filgotinib in IIM, selective JAK1 inhibition may show considerable efficacy in the management of IFN-I mediated manifestations, such as cutaneous and muscular involvement in DM patients. However, in light of the complexity of intracellular signaling pathways these considerations are purely speculative and still await confirmation by solid evidences coming from clinical experiences and trials.

## Author contributions

GS and GL conceived the manuscript. GS, GL, DS, AL, and FF wrote the manuscript. CV, CB, SP, MC, and LM critically revised the manuscript. All authors provided approval for publication of this content and agreed to be accountable for all aspects of the work in ensuring that questions related to the accuracy or integrity of any part of the work are appropriately investigated and resolved and participated in the selection of articles and their interpretation.
